# Pyrite-based denitrification combined with electrochemical disinfection to remove nitrate and microbial contamination from groundwater

**DOI:** 10.1038/s41545-023-00269-3

**Published:** 2023-08-24

**Authors:** Eleftheria Ntagia, Piet Lens

**Affiliations:** National University of Ireland, Galway, University Road, H91 TK33 Galway, Ireland

**Keywords:** Pollution remediation, Sustainability

## Abstract

Nitrate and microbial contamination of groundwater can occur in countries that face intense urbanization and inadequate sanitation. When groundwater is the main drinking water source, as is often the case in such countries, the need to remove these contaminants becomes acute. The combination of two technologies is proposed here, a biological step to denitrify and an electrochemical step to disinfect the groundwater, thereby aiming to reduce the chemical input and the footprint of groundwater treatment. As such, a pyrite-based fluidized bed reactor (P-FBR) was constructed to autotrophically denitrify polluted groundwater. The P-FBR effluent was disinfected in an electrochemical cell with electrogenerated Cl_2_. Nitrate was removed with 79% efficiency from an initial 178 mg NO_3_^−^ L^−1^ at an average denitrification rate of 171 mg NO_3_^−^ L^−1^ d^−1^, with 18 h hydraulic retention time (HRT). The electrochemical unit achieved a 3.8-log reduction in total coliforms with a 41.7 A h m^−3^ charge density.

## Introduction

Groundwater constitutes the main potable water source in low-income countries, greatly in Sub-Saharan countries, where the share for agricultural and industrial sectors is low, as well as in countries such as Ireland and the UK where these sectors are primarily rainfed^1,2^. The main pressors for groundwater quality deterioration are poor sanitation facilities, uncontrolled release of ailing treated domestic and industrial wastewater, cattle farming with uncontrolled manure spreading and intense fertilizing activities. Local conditions can intensify groundwater pollution, such as vulnerable aquifers and the intensification of extreme weather conditions^1,3–5^.

Elevated concentrations of nitrate (NO_3_^−^), chloride (Cl^−^), as well as microbial indicators such as fecal and total coliforms (TC) suggest anthropogenic groundwater pollution. Nitrate concentrations as high as 500 mg NO_3_^−^ L^−1^ have been reported in peri-urban areas of low-income countries, alongside with 300 mg Cl^−^ L^−1^ and 2 log CFU 100 mL^−1^ *Escherichia coli* (*E. coli*) and 4 log CFU 100 mL^−1^ TC^4–6^. The concentrations vary largely with the local geological conditions and with the source of contamination. As a way to tackle waterborne diseases, the WHO has set a maximum of 50 mg NO_3_^−^ L^−1^ and 0 CFU 100 ml^−1^ for TC in drinking water^7^.

Groundwater is poor in organic content, therefore conventional treatment schemes including heterotrophic denitrification are not financially sustainable. An option is to target autotrophic groundwater denitrification with electron donors such as hydrogen gas (H_2_), elemental sulfur (S^0^), sulfide (HS^−^), thiosulfate (S_2_O_3_^2−^), ferrous iron (Fe^2+^) or even pyrite (FeS_2_)^8,9^. Based on the respective stoichiometric equations the denitrification capacities range from 2.5 g NO_3_^−^-N g^−1^ e^−^ donor for H_2_ to 0.05 and 0.1 g NO_3_^−^-N g^−1^ e^−^ donor for reduced iron (Fe^2+^ and Fe^0^)^9,10^. Pyrite driven denitrification occurs naturally in aquifers^11–14^ and FeS_2_ oxidation is coupled to microbial NO_3_^−^ reduction to nitrogen gas (N_2_), with a 3 mol NO_3_:1 mol FeS_2_ stoichiometric ratio, accompanied by the production of 2 mol SO_4_^2−^ ^12,15^. Furthermore, FeS_2_ has been utilized in low C/N wastewater treatment^16,17^, as well as in groundwater denitrification, mainly in bottle tests^18,19^. Pyrite is a ubiquitous, low-cost mineral that is frequently found as a waste product of mining activities^20^. Additionally, during FeS_2_ denitrification a circum-neutral pH is maintained, resulting in minimization of chemical inputs for this process^17,18^.

In addition to denitrification, polishing steps are required to provide safe drinking or irrigation water, as the microbial load of the denitrified effluent, will not allow for direct consumption or reuse^21^. Several disinfection practices have been used so far to polish water treatment effluents and chemical oxidation, either with free chlorine, ozone (O_3_) or UV, is generally preferred for potable water reuse^22^. However, alternative disinfection methods are being sought to provide the treatment process with chemical and grid independency, including electrochemical disinfection, also referred to as electrochlorination when free chlorine is the chemical agent produced^23^. In electrochlorination, chloride ions (Cl^−^) naturally contained in groundwater are oxidized to chlorine (Cl_2_) at the surface of an electrode, when a constant source of current is applied by an external power source^24,25^. The produced Cl_2_ mixes with the bulk electrolyte and undergoes hydrolysis, producing two potent disinfectants: hypochlorous acid (HOCl) and hypochlorite (OCl^−^). The ratio of the two is determined by the solution pH^26,27^. Electrochemical disinfection has been tested for directly treating groundwater or irrigation water contaminated with pathogens^28–30^, but also as a polishing step for bioreactor effluents^27,31^. The disinfection efficiency will depend upon the anode material, the Cl^−^ concentration of the electrolyte, the pH and upon the organics and ammonia content of the water to be treated^22^.

This study combined continuous autotrophic denitrification with FeS_2_ as electron donor and electrochemical disinfection through Cl^−^ oxidation to Cl_2_ (HOCl + OCl^−^) to treat groundwater. Here, polluted groundwater was treated in a pyrite-based fluidized bed reactor (P-FBR) and the P-FBR effluent was disinfected through chlorination in the anodic compartment of the electrochemical cell. The efficiency of FeS_2_-based autotrophic denitrification at ambient conditions and at high Cl^−^ concentrations was investigated aiming for long-term FeS_2_ denitrification of real groundwater. Furthermore, the electrochlorination efficiency with Pt/Ti electrodes and the effect of the real groundwater matrix on electrochlorination were studied. The electrochemical disinfection efficiency of the FeS_2_ denitrified effluents was finally tested.

## Results

### Continuous denitrification of synthetic groundwater in the pyrite-based fluidized bed reactor (P-FBR)

**Fig. 1:**
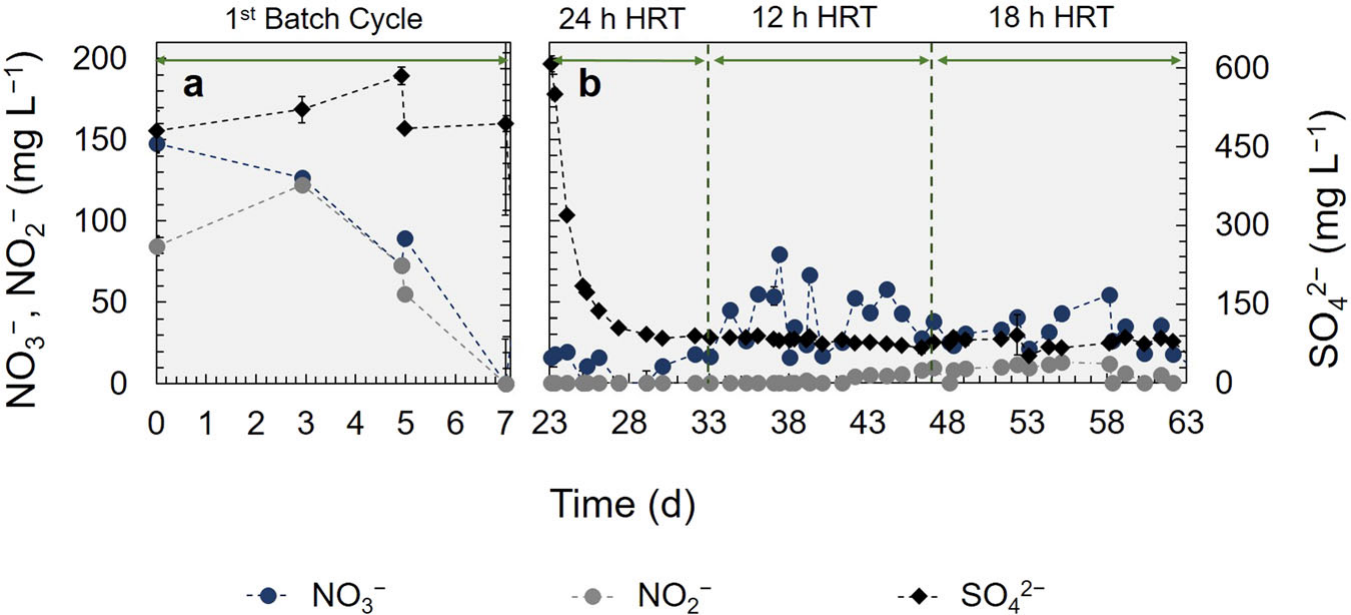
Total operational period of the P-FBR denitrification reactor with synthetic polluted groundwater (SGW). **a** First batch operational cycle (days 0–7) and **b** continuous operation (days 23–64) with 24 h, 12 h and 18 h HRT. The error bars represent the standard deviation of *n* = 3 analytical replicates.

The biological step was a denitrification FBR with a pyrite (FeS_2_) bed as electron donor (P-FBR). The P-FBR was inoculated and operated in batch with synthetic polluted groundwater (SGW) until NO_3_^−^ was eliminated (details provided in the Methods section). The first batch lasted seven days (Fig. 1) and 44 mg NO_3_^−^ L^−1^ d^−1^ was the highest nitrate removal rate achieved. Within the first three days, the NO_3_^−^ decrease was accompanied by a NO_2_^−^ increase, which was then consumed, indicating complete denitrification of NO_3_^−^ to N_2_ gas. Between days 7 and 23, several batch cycles took place to determine the denitrification rates and determine the hydraulic retention time (HRT) for the continuous operation (data not shown).

Starting on day 23, the P-FBR was operated in continuous mode for 41 days with SGW (Fig. 1). During this operation three different HRTs were tested, 24, 12 and 18 h, aiming to both achieve an effluent NO_3_^−^ concentration lower than 50 mg NO_3_^−^ L^−1^ and also to minimize the groundwater treatment time. The denitrification rate achieved at 24 h HRT was 167.9 (±7.5) mg NO_3_^−^ L^−1^ d^−1^ (94.3 ± 4.2% NO_3_^−^ removal efficiency) and the effluent NO_3_^−^concentration was 10.1 (±7.5) mg NO_3_^−^ L^−1^ (Table 1). When the HRT was decreased to 12 h, the NO_3_^−^ removal rate decreased to 93.2 (±37.0) mg NO_3_^−^ L^−1^ d^−1^ (76.2 ± 10.4% NO_3_^−^ removal efficiency) and the NO_3_^−^ effluent concentration increased to 42.4 (±18.5) mg NO_3_^−^ L^−1^. During that period, NO_2_^−^ started accumulating, up to 6.4 (±8.4) mg NO_2_^−^ L^−1^, signifying incomplete denitrification due to the increased NO_3_^−^ loading rate at the decreased HRT. At HRT 18 h, denitrification rates resumed to an average 134.7 (±14.1) mg NO_3_^−^ L^−1^ d^−1^ (81.8 ± 6.0% NO_3_^−^ removal efficiency), with 32.5 (±10.6) mg NO_3_^−^ L^−1^ effluent concentrations, always below the maximum allowed drinking water levels^7^. Higher NO_2_^−^ concentrations were measured during that period, on average 22.0 (±15.1) mg NO_2_^−^ L^−1^. However, this could be attributed to the 12 h HRT operation effect, which is also confirmed by the absence of NO_2_^−^ in the subsequent operational periods with real groundwater. The pH remained stable at 7 at all three HRT tested with SGW (7.3 ± 0.1, 7.1 ± 0.1 and 7.0 ± 0.0 at respectively 24, 12 and 18 h HRT), without chemical dosing for pH control.

### Continuous denitrification of real groundwater in the pyrite-based fluidized bed reactor (P-FBR)

After establishing a stable, continuous P-FBR operation with SGW at 18 h HRT, the influent was switched to real groundwater (GW) and the P-FBR was operated for 35 days, completing three treatment cycles with GW with different Cl^−^ and TC loads (Table 2, Methods section). During these 35 days of operation with GW a stable denitrification performance was maintained with an average denitrification rate of 171 mg NO_3_^−^ L^−1^ d^−1^ (average NO_3_^−^ loading rate was 215 mg NO_3_^−^ L^−1^ d^−1^) for all three cycles, corresponding to an average 79% denitrification efficiency for the three cycles with GW (Table 1). Accordingly, the average effluent NO_3_^−^ concentration was 33 mg NO_3_^−^ L^−1^, meeting the standard for reuse as potable water and furthermore, NO_2_^−^ was not detected at any of the GW operational cycles.

**Table 1:** Denitrification efficiency of the pyrite-based fluidized bed reactor (P-FBR) during continuous operation with synthetic (SGW) and real (GW) groundwater.

Operational cycle	HRT [h]	Effluent concentrations [mg L^−1^]				NO_3_^−^ removal efficiency [%]	NO_3_^−^ removal rate [mg NO_3_^−^ L^−1^ d^−1^]	Effluent electrical conductivity [mS cm^−1^]
		NO_3_^−^	NO_2_^−^	Cl^−^	SO_4_^2−^			
SGW-Cycle I	24	10.1 ± 7.5	0.0 ± 0.0	345.9 ± 36.5	99.8 ± 17.6	94.3 ± 4.2	167.9 ± 7.5	2.1 ± 0.3
SGW-Cycle II	12	42.4 ± 18.5	6.4 ± 8.4	293.4 ± 40.2	80.0 ± 6.1	76.2 ± 10.4	93.2 ± 37.0	2.0 ± 0.1
SGW-Cycle III	18	32.5 ± 10.6	22.0 ± 15.1	274.3 ± 29.0	77.0 ± 10.6	81.8 ± 6.0	134.7 ± 14.1	1.9 ± 0.1
GW-Cycle I	18	24.1 ± 8.3	0.8 ± 2.3	93.0 ± 9.4	27.8 ± 4.8	85.5 ± 5.3	175.6 ± 27.7	0.9 ± 0.0
GW-Cycle II	18	40.6 ± 8.1	0.0 ± 0.0	268.2 ± 41.0	26.9 ± 1.5	76.6 ± 8.1	163.8 ± 22.4	1.8 ± 0.2
GW-Cycle III	18	33.7 ± 18.6	0.0 ± 0.0	275.7 ± 75.5	28.1 ± 2.6	75.7 ± 10.3	173.4 ± 30.3	2.7 ± 0.9

**Fig. 2:**
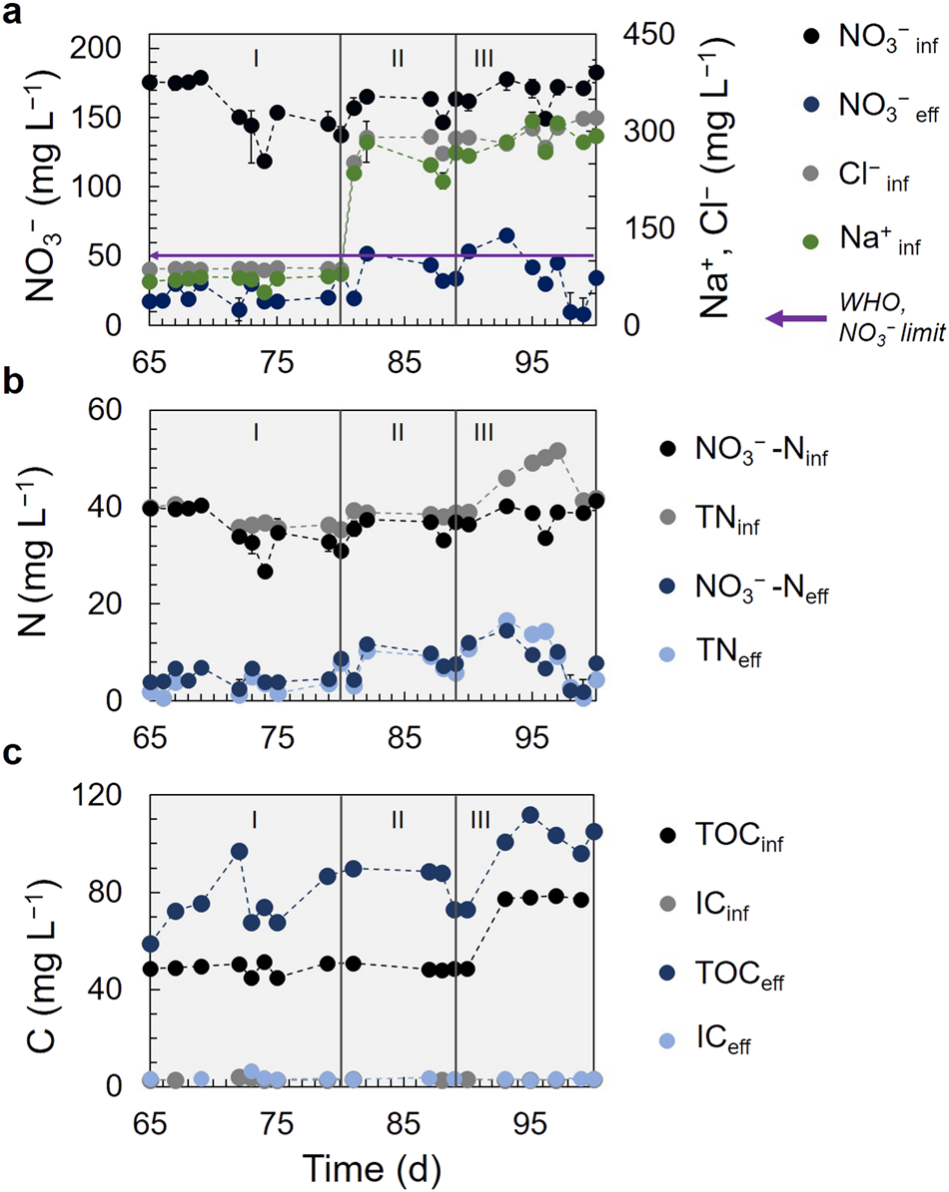
P-FBR operational performance with real groundwater (GW) (days 65–100). **a** NO_3_^−^ removal and changes after Cl^−^ and Na^+^ increase observed in GW-Cycles II and III, **b** total nitrogen (TN) and nitrate nitrogen (NO_3_^−^-N) and **c** total organic carbon (TOC) and inorganic carbon (IC), in the influent and effluent of the P-FBR. The error bars represent the standard deviation of *n* = 3 analytical replicates.

Although the denitrification rates along the three cycles with GW did not present significant changes, a marginal decrease in the denitrification rate, alongside with a marginal increase in the effluent NO_3_^−^ concentration were observed after day 80 (Fig. 2a and Table 1). On day 80, the GW-Cycle II started, where the Cl^−^ concentration was increased from 93 to 268 mg Cl^−^ L^−1^, similar to the initial operation with SGW (Fig. 2a and Table 1), accompanied with an increase in the Na^+^ concentration from an average 77 to 234 mg Na^+^ L^−1^. This greater concentration of NaCl negatively affected the denitrifying activity in the P-FBR, however, denitrification was not inhibited and after 15 days of operation at higher Cl^−^ concentrations (day 95 in Fig. 2a), the performance recovered with a NO_3_^−^ effluent concentration below the limit of 50 mg NO_3_^−^ L^−1^.

The GW only contained a low amount of ammonium nitrogen, as almost all of the total nitrogen (TN) was measured as NO_3_^−^-N in both the influent and the effluent of the P-FBR (Fig. 2b). However, the GW presented a higher organic load, 48.7 (±2.8) mg TOC L^−1^ for GW-Cycle I, 48.3 (±0.3) mg TOC L^−1^ for GW-Cycle II and 77.7 (±0.3) mg TOC L^−1^ for GW-Cycle III (Fig. 2c), compared to SGW that had no organic content. In GW-cycle III, this higher TOC load can also partially be attributed to the higher TC load, 500 MPN 100 mL^−1^, compared to 7 and 2 MPN 100 mL^−1^ in GW-Cycles I and II, naturally contained in the GW and no microbial load in the SGW. The TOC concentration was consistently higher in the effluent than in the influent, 77.2 (±10) mg TOC L^−1^ in GW-Cycle I, 83.2 (±7.3) mg TOC L^−1^ in GW-Cycle II and 103.5 (±5.2) mg TOC L^−1^ in GW-Cycle III, indicating a release of organic matter in the denitrified groundwater.

The SO_4_^2−^ concentration was marginally higher in the effluent than in the influent, except for GW-Cycle III. The influent SO_4_^2−^ concentration was 25.9 (±3.4) mg L^−1^, 25.7 (±0.6) mg L^−1^ and 30.4 (±0.6) mg L^−1^ and the effluent SO_4_^2−^ concentration was 27.8 (±4.8) mg L^−1^, 26.9 (±1.5) mg L^−1^ and 28.1 (±2.6) mg L^−1^, in GW-Cycles I, II and III, respectively (Supplementary Table 1). The concentration of all cations remained unchanged between the influent and the effluent of the P-FBR (Supplementary Table 2), apart from potassium (K^+^). The influent K^+^ concentration in GW-Cycle I and II was close to and below the ICP detection limit, respectively, whilst in GW-Cycle III it was 3.6 (±0.5) mg L^−1^. However, in the effluent, 2.1 (±0.9), 1.2 (±0.6) and 5.4 (±1.0) mg K^+^ L^−1^ were detected in GW-Cycles I, II and III, respectively, (Supplementary Table 2 and Supplementary Fig. 1), consistently higher than the K^+^ in the P-FBR influent. The pH remained stable at 7.3 during GW treatment without any chemical addition for pH control. The Cl^−^ concentration, essential for the subsequent disinfection step, remained also unchanged between influent and effluent of the P-FBR (Table 1). Small changes in the organic content, the concentration of anions and cations or the pathogens load between the influent and effluent of the P-FBR did not affect the conductivity and resulted in an effluent conductivity of 1.8 (±0.2) and 2.7 (±0.9) mS cm^−1^, for GW-Cycles II and II, respectively (Table 1 and Supplementary Table 1).

### Electrochlorination efficiency evaluated with denitrified groundwater

**Fig. 3:**
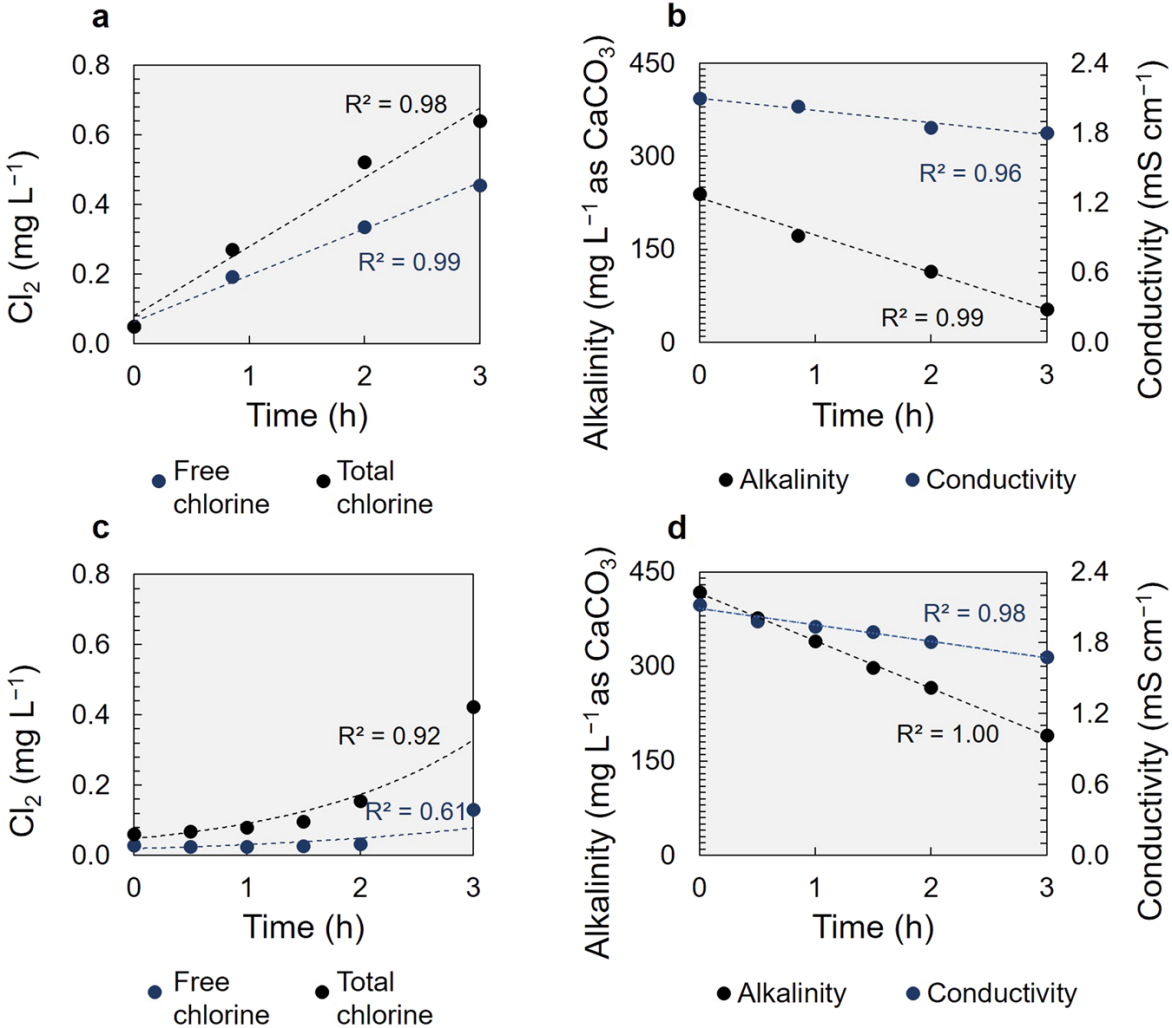
Synthetic (SGW) vs real groundwater (GW-Cycle III P-FBR effluent) electrochlorination at 50 mA current (*j* = 2 mA cm^−2^ current density). **a** SGW chlorine, **b** SGW alkalinity and conductivity, **c** GW chlorine and **d** GW alkalinity and conductivity. The error bars represent the standard deviation of *n* = 3 analytical replicates.

Batch, three-hour electrolysis experiments were conducted at 50 mA constant current (*j* = 2 mA cm^−2^) to investigate Cl_2_ evolution at the Pt/Ti anode with the SGW P-FBR effluent (Fig. 3a, b) and the GW-Cycle III P-FBR effluent (Fig. 3c, d). The initial Cl^−^ concentration for the two tests was similar, i.e. 289 (±6.5) mg Cl^−^ L^−1^ and 295.2 (±0.9) mg Cl^−^ L^−1^ for SGW and GW, respectively, while the two effluents differed in initial TOC concentration. This resulted in differences in measurable free and total chlorine as well as in the calculated respective chlorine production rates (Fig. 3a, c). Chlorine was produced with SGW at 1.65 (±0.05) V vs Ag/AgCl anode potential and with GW-Cycle III effluent at 1.43 (±0.01) V vs Ag/AgCl anode potential.

During constant current electrolysis with SGW the free and total chlorine increased with run time resulting in 0.455 (±0.002) mg L^−1^ final free chlorine concentration and 0.640 (±0.005) mg L^−1^ total chlorine. The production rate obtained was 3.89 mg L^−1^ d^−1^ free chlorine and 5.66 mg L^−1^ d^−1^ total chlorine (Fig. 3a). The production rates remained stable between the first and second hour of operation, at 4.12 and 4.09 mg L^−1^ d^−1^ for free chlorine and 6.37 and 7.24 mg L^−1^ d^−1^ for total chlorine for the first and second hour, respectively, while they dropped in the last hour to 3.46 mg L^−1^ d^−1^ free and 3.38 mg L^−1^ d^−1^ total chlorine. The pH dropped from initially pH 7.90 to 6.74 at which 85% of the free chlorine is present as HOCl and the remaining as OCl^−26^. Similarly, the alkalinity and conductivity decreased from an initial 239.4 (±3.7) mg L^−1^ as CaCO_3_ to a final 53.9 (±1.2) mg L^−1^ as CaCO_3_ and from initially 2.1 (±0.0) mS cm^−1^ to a final conductivity of 1.8 (±0.0) mS cm^−1^ (Fig. 3b).

After the three-hour GW-Cycle III effluent test, the measured free chlorine concentration was 0.132 (±0.001) mg L^−1^ and the total chlorine was 0.423 (±0.003) mg L^−1^. The production rate for free chlorine was of 0.56 mg L^−1^ d^−1^ and for total chlorine, it was 2.12 mg L^−1^ d^−1^ (Fig. 3c). The pH dropped from initially 7.96 to 6.83. Accordingly, the alkalinity and conductivity decreased from an initial 418.3 (±4.3) mg L^−1^ as CaCO_3_ to a final 187.9 (±4.1) mg L^−1^ as CaCO_3_ and from an initial 2.1 (±0.0) mS cm^−1^ to a final 1.7 (±0.0) mS cm^−1^ conductivity (Fig. 3d).

### Electrochemical disinfection of denitrified groundwater and the residual chlorine effect

**Fig. 4:**
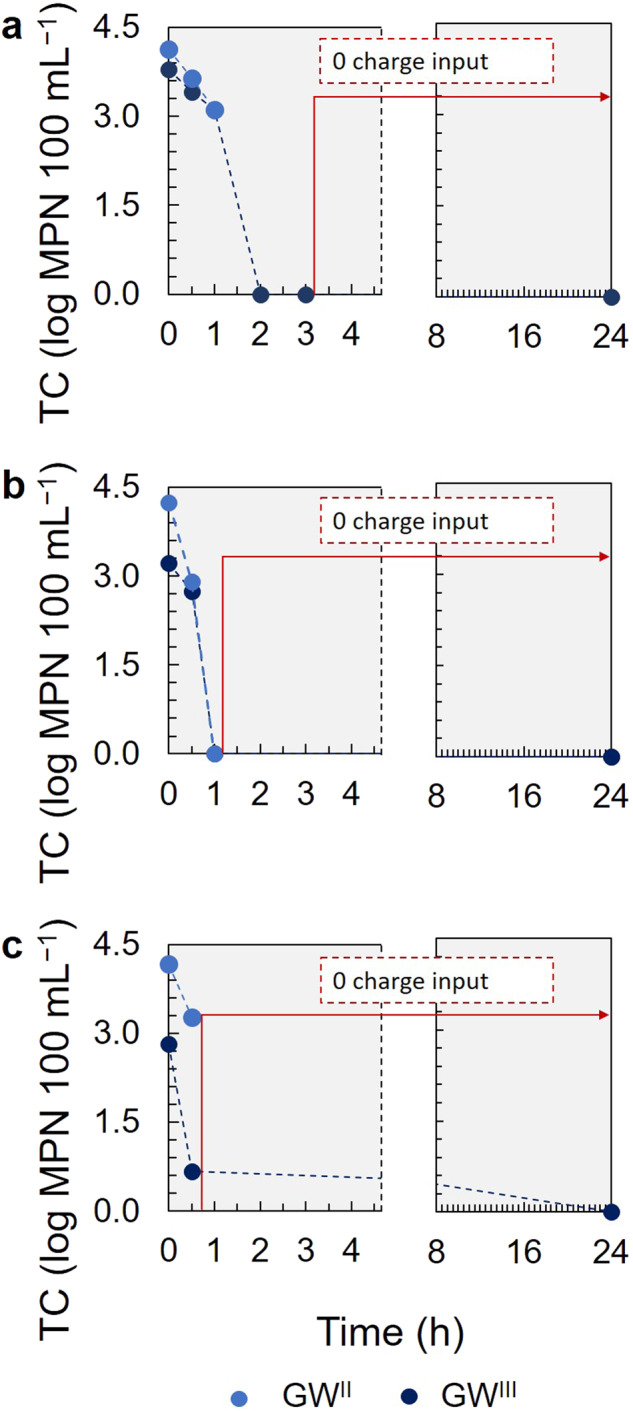
Disinfection of real groundwater (GW) at 50, 100 and 150 mA applied current (*j* = 2 mA cm^−2^, 4 mA cm^−2^ and 6 mA cm^−2^). **a**: 50 mA, **b**: 100 mA and **c**: 150 mA, in two replicates, conducted with the P-FBR GW-Cycle II (light blue circles) and GW-Cycle III (dark blue circles) effluents, respectively. The residual effect of free chlorine is demonstrated at 24 h TC counts.

The disinfection efficiency was further investigated in the presence of TC with the P-FBR GW-Cycles II and III effluents during electrolysis at 50, 100 and 150 mA (*j* = 2 mA cm^−2^, 4 mA cm^−2^ and 6 mA cm^−2^) (Fig. 4). To assess the impact of residual free chlorine, the TC load was measured in the disinfected water 24 hours after the completion of electrolysis.

At 2 mA cm^−2^ and with GW-Cycle II effluent, the free and total chlorine produced within 1 h were 0.049 (±0.002) mg L^−1^ and 0.109 (±0.001) mg L^−1^, respectively, and the final pH was 7.07. During this run the TC load decreased by 1.01 log to a final 3.12 log MPN 100 mL^−1^ (Fig. 4a). In the run with GW-Cycle III effluent, completed in 3 h, the TC counts were eliminated already in 2 h of applied electrolysis time, achieving a 3.79 log removal of TC (Fig. 4a) when the free and total chlorine were 0.032 (±0.003) mg L^−1^ and 0.155 (±0.002) mg L^−1^, respectively. At the completion of this test, *t* = 3 h, the final free and total chlorine concentrations were 0.132 (±0.001) mg L^−1^ and 0.423 (±0.003) mg L^−1^, respectively and the final pH 6.84. At that time the cell was left in open circuit and the residual chlorine effect was demonstrated after 24 h. No TC could be detected in the disinfected groundwater (Fig. 4a).

At 4 mA cm^−2^, the TC load was eliminated within 1 h in both GW-Cycle II and III, with 4.24 and 3.22 log removal for GW-Cycle II and III, respectively (Fig. 4b). The measured free and total chlorine varied in this case between the two cycles, with final 0.265 (±0.000) mg L^−1^ and 0.059 (±0.004) mg L^−1^ free chlorine concentration and 0.449 (±0.001) mg L^−1^ and 0.261 (±0.004) mg L^−1^ total chlorine concentration for GW-Cycle II and III, respectively. The final pH was 6.84 and 7.03 in GW-Cycle II and III.

At 6 mA cm^−2^ GW-Cycle II effluent, a 0.9 log TC removal was achieved within 30 min electrolysis time with a final 3.27 log MPN 100 mL^−1^ TC concentration, while for GW-Cycle III, the TC load decreased by 2.15 log, obtaining a treated groundwater with a 0.67 log MPN 100 mL^−1^ TC load (Fig. 4c). The measured free and total chlorine varied as well in this case between the two cycles, with 0.197 (±0.003) mg L^−1^ and 0.036 (±0.002) mg L^−1^ final free chlorine and 0.273 (±0.003) mg L^−1^ and 0.150 (±0.005) mg/L total chlorine concentrations for GW-Cycle II and III, respectively. These differences in chlorine concentration measurements between cycles II and III could have been resulting from differences in the local mixing conditions or differences in organics or metals present in the GW-Cycle III effluent that could interfere with the chlorine analysis. While the TC counts at 6 mA cm^−2^ GW-Cycle III were not completely eliminated within the electrolysis time, no TC were detected after 24 h.

### The effect of charge density on energy consumption and disinfection

**Fig. 5:**
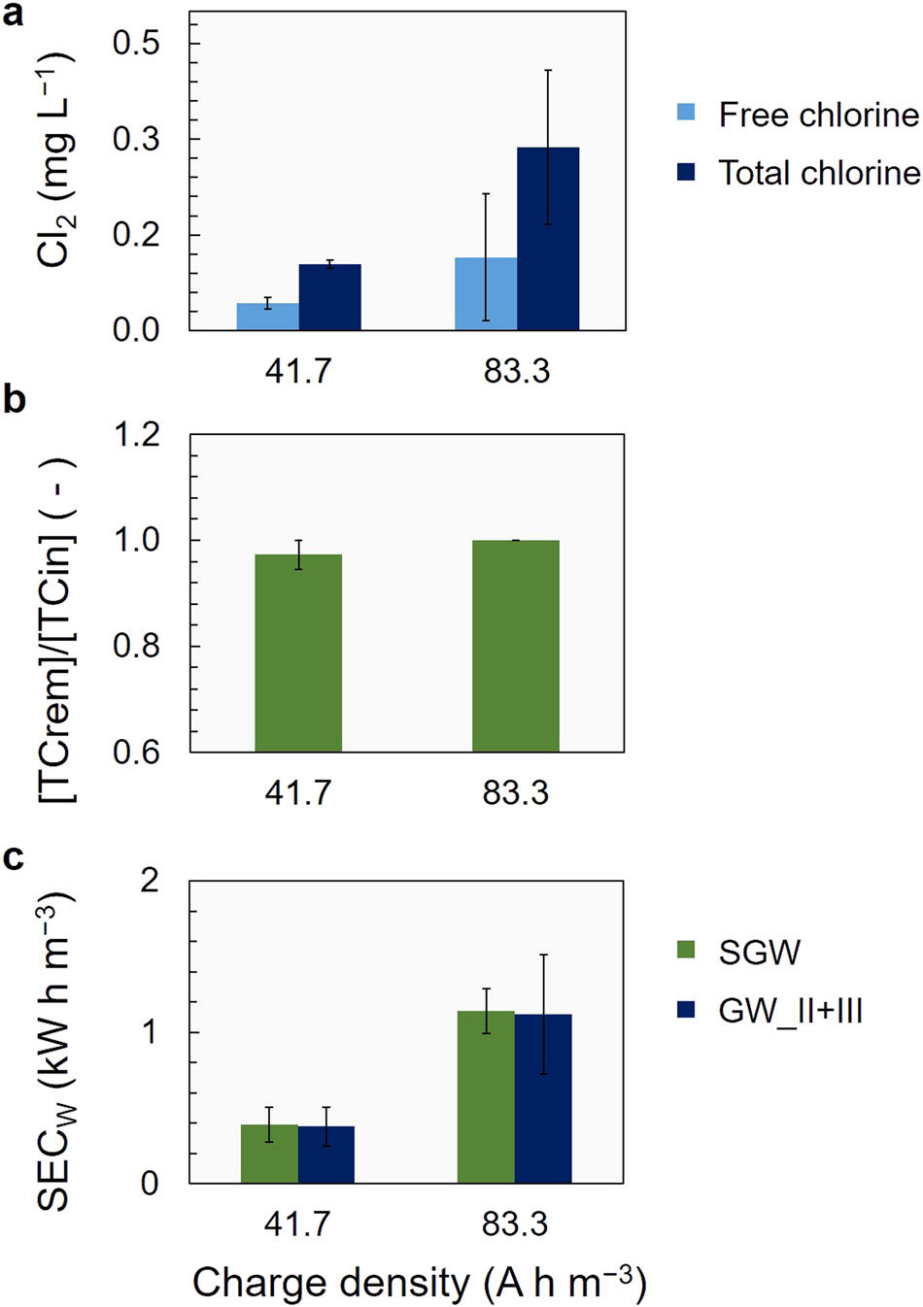
Charge density effect on the efficiency of disinfection and on the energy consumption. **a** Chlorine (free and total) production (in mg L^−1^), **b** total coliforms (TC) removal expressed as the ratio of log TC removal and initial TC log concentration ((log MPN 100 mL^−1^)/(log MPN 100 mL^−1^)) and **c** specific energy consumption for water treatment (SEC_W_ in (kW h m^−3^) for synthetic (SGW) and real groundwater from Cycles II and III (GW_II + III) at 41.7 and 83.3 (A h m^−3^) charge density. The error bars represent the standard deviation of *n* = 3 experimental runs with GW-Cycle II and III and *n* = 4 experimental runs with SGW.

Two charge densities, 41.7 and 83.3 A h m^−3^, were selected to investigate the impact of increasing charge density on chlorine production and disinfection efficacy. Additionally, the specific energy consumption of the electrochemical treatment was assessed. Both free and total chlorine increased with the charge density (Fig. 5a), however, the difference in the two concentrations obtained was not statistically significant. Considering that the denitrified groundwater is an impure electrolyte, differences in the TOC, the microbial load or even background color can induce variations in the production and measurement of Cl_2_. Thus, it is important to exercise caution when comparing the two charge densities in terms of free chlorine production with denitrified groundwater, or in general polluted water, as the electrolyte.

A lower charge density and a lower chlorine concentration were already sufficient for complete TC elimination (Fig. 5b). More specifically, a 0.97 (±0.03) ratio of log TC decrease over the initial log TC counts was achieved with 41.7 A h m^−3^ and 0.043 (±0.009) mg L^−1^ free chlorine. Complete elimination of the TC counts was achieved with 83.3 A h m^−3^ and 0.115 (±0.099) mg L^−1^ final free chlorine (Fig. 5a, b). Consistently lower values of free and total chlorine were measured with the GW compared to the SGW. Therefore, for a fair comparison, the free chlorine produced with SGW was 0.169 (±0.037) mg L^−1^ and 0.245 (±0.065) mg L^−1^ with 41.7 A h m^−3^ and 83.3 A h m^−3^, respectively, while the total chlorine for the same charge densities was 0.300 (±0.006) mg L^−1^ and 0.471 (±0.121) mg L^−1^, respectively (Supplementary Table 3).

The specific energy consumption (SEC_W_) for the disinfection of the denitrified groundwater increased with increasing charge density, both for SGW and GW (Fig. 5c). The SEC_W_ was ~0.4 and ~1.1 kW h m^−3^ for both SGW and GW at 41.7 and 83.3 A h m^−3^, respectively, and the similarity can be attributed to the similar conductivities of the two solutions (~2 mS cm^−1^). At 41.7 A h m^−3^, the cell voltage with SGW was 10.9 (±1.7) V and with GW, 9.1 (±3.1) V. At 83.3 A h m^−3^ the cell voltage obtained was 13.7 (±1.8) V and 13.4 (±4.7) V, respectively.

## Discussion

The average denitrification rate obtained by the P-FBR was 171 mg NO_3_^−^ L^−1^ d^−1^ at 22 (±2) °C, 1 to 2 orders of magnitude higher than the rates obtained in bottle studies on FeS_2_-based groundwater denitrification^18,19^. To date, the majority of research on autotrophic FeS_2_ denitrification has been carried out at around 30 °C, as this temperature range is expected to facilitate higher denitrification activity. The effect of temperature on the denitrification efficiency was studied by Xu et al.^32^, who demonstrated that a drop of temperature from 28 to 20 °C resulted in a 48% decrease in the denitrification efficiency, while it also contributed to an increase in the effluent NO_2_^−^ concentration. One of the few studies conducted at 20 °C was the study of Tong et al.^33^, where a 56.4% NO_3_^−^ removal efficiency was achieved, with a similar sized column as in the present study, however with a three times higher FeS_2_ mass.

In our study, low effluent SO_4_^2−^ concentration was obtained, 28 mg L^−1^ in average for all three cycles with real groundwater (Table 1), with a marginal increase of the SO_4_^2−^ concentration from the influent to the effluent of the P-FBR (Table 1 and Supplementary Table 1). An increase in SO_4_^2−^ as a product of FeS_2_-based denitrification is expected, however, the produced SO_4_^2−^ was well below the expected stoichiometric amount based on the NO_3_^−^ removal. Consistently lower SO_4_^2−^ concentrations than the stoichiometrically expected have been reported in FeS_2_ denitrification studies, without providing conclusive, quantitative evidence^33–35^. Although the low SO_4_^2−^ effluent concentrations obtained seem advantageous for water reuse schemes, longer-term experiments and a more detailed examination of the denitrification products and the precipitates formed in FeS_2_-based denitrification reactors will be required to conclude on this described advantage of FeS_2_ over other sulfurous electron donors^33^.

Furthermore, a marginally higher K^+^ concentration was observed in the effluent of the denitrifying reactor in our study that was consistent in all three cycles with GW and cannot be attributed to any specific reactions describing FeS_2_ denitrification. It can be hypothesized that the K^+^ measured in the effluent originated from the FeS_2_ as an impurity and was subsequently released after FeS_2_ utilization. In a study conducted on FeS_2_ aerobic bioleaching the initial FeS_2_ used contained K, while the neutralizing agent not. The elemental composition of the bioleach liquor consistently showed a K concentration higher than 1% after pyrite biooxidation^36^. While this suggests the possibility of K^+^ leaching following FeS_2_ biooxidation, there is no conclusive evidence to support the hypothesis that the higher K^+^ levels in our effluent are a result of this process, particularly since the experimental conditions in that study, specifically the pH and oxidative conditions, were different from those in our experiments. Moreover, we did not conduct a FeS_2_ compositional analysis. Hence, the hypothesis that K^+^ in the effluent originated from the impurities present in FeS_2_ deserves further scrutiny. In conclusion, it is crucial to thoroughly examine the composition of FeS_2_ before use, particularly in water reuse schemes.

The ability of bacterial cells to use FeS_2_, a solid electron donor, for denitrification has been demonstrated in nature, yet the exact mechanism of the FeS_2_ utilization is still being debated^10^. Direct FeS_2_ utilization or indirect, through FeS_2_ dissolution mechanisms, have been proposed. However, it is difficult to determine which is the most probable route and to distinguish between the two^10,37^. The high denitrification rates obtained in this study could be partially attributed to the active denitrifying mixed community present in the biomass that was used to inoculate the P-FBR, which was obtained from the study of Carboni et al.^17^. Furthermore, between batch and continuous operation (day 8) the O_2_ content of the headspace increased from between 2 to 3% up to 10% due to an operational upset (Supplementary Fig. 2a). This resulted in a measurable increase in the Fe^2+^ and S_2_O_3_^2−^ concentrations in the reactor (Supplementary Figs. 2a and 2b), which could be linked to FeS_2_ dissolution^10^. After this upset, denitrification rates as high as 400 mg NO_3_^−^ L^−1^ d^−1^ were observed, around ten times higher than in the first batch operation and the subsequent continuous operation. Previous research on FeS_2_ autotrophic denitrification has suggested that FeS_2_ dissolution might be an essential step prior to its utilization for denitrification^15,38–40^. This suggests that the previously mentioned mechanism may have occurred in the reactor.

However, these results are rather limited to allow for commentary on the exact mechanism of FeS_2_ obtained in the studied reactor during the total course of the experimental period. Surface and composition analysis of the FeS_2_ before and after operation could reveal changes in the morphology of the FeS_2_ and give an indication of the parts of FeS_2_ utilized by the microbial community for denitrification^37^. Studies that combine microbial community analysis with surface analysis in controlled media are essential in deciphering the FeS_2_-driven denitrification mechanisms. The latter will also allow for future optimization of FeS_2_ autotrophic denitrification systems and for establishment of these systems in the water treatment lines.

Chlorine production was achieved with a Pt/Ti anode and efficiently disinfected the denitrified SGW and GW effluents (Fig. 3). In electrochlorination studies, electrodes employing expensive, mixed metal oxide (MMO) coatings^27,41,42^ or boron-doped diamond (BDD)^28^ electrodes have been extensively tested, as they are more robust and demonstrate higher oxidation rates. More specifically, Ru MMO demonstrates a higher affinity towards Cl_2_ evolution^23,43^, compared to O_2_ evolution, two reactions that occur at similar electrode potentials^24,25^. In this study, a custom-made Pt/Ti electrode with a Ti current collector laser welded perpendicularly to the electrode surface was used as an anode and supported complete disinfection of real GW (Fig. 4 and Supplementary Fig. 3c).

At 41.7 A h m^−3^ a 3.8 log removal of TC was achieved corresponding to a 0.4 kW h m^−3^ SECw, when the free chlorine measured was in average 0.043 mg L^−1^ with GW and 0.169 mg L^−1^ with SGW. Patermarakis and Fountoukidis^44^ in one of the first studies on electrochlorination with a low-cost Ti electrode achieved in average a 5-log removal of germs, without specifying the type, with 89.3 A h m^−3^ charge investment, resulting in 4 kW h m^−3^ SECw. A Pt/Ti anode was also used in the study of Qing et al.^30^ that reduced the microbial content of irrigation water by 5 log with 9 A h m^−3^ charge investment and achieved a three times higher free chlorine concentration, even though the initial Cl^−^ concentration was as low as 1.85 mg/L. In their study to treat microbially contaminated groundwater, De Battisti et al.^28^ used a BDD electrode that eliminated the 200 MPN 100 mL^−1^ TC and *E. coli* load with a 28 A h m^−3^ charge density, however, no results were reported on the cell voltage obtained to allow for SECw calculations. Disinfection was evaluated in our study using total coliforms (Fig. 4). However, more persistent pathogens, including viral species, might require higher doses of chlorine and longer contact times. Thus, further research on electrochlorination times and power investment for those microbial contaminants should be conducted^28,45,46^.

The production rates for both free and total chlorine with SGW remained stable between the first and the second hour of operation, while they dropped in the last hour (Fig. 3). Several processes could be responsible for this reduced Cl_2_ production rate in our system. For instance O_2_ bubble formation, which was apparent in our experiments, obstructing the electrolyte-electrode contact and thus limiting the Cl^−^ oxidation rate, electrode surface modifications induced by oxidation reactions, or further, free chlorine inorganic by-products formation^25,44,47^. Without specific analysis of all the chlorination products produced, the pH changes in the electrolyte and without electrode surface analysis before and after the experimental cycles, it is difficult to identify which specific process, or combination of processes, is responsible for the observed effects.

Regardless of the mechanism that led to the reduction of chlorination rates in the last hour of the experiment, what is most important in the case of disinfection is maintaining consistency and a stable voltage to ensure effective treatment. One way to achieve this may be through reverse polarity^30,44^. To ensure long-term operational stability of the electrodes for disinfection, further testing is required. Additionally, careful examination of the by-products resulting from Cl_2_ disinfection is necessary when considering water reuse. Prioritizing these factors is essential to ensure that the proposed disinfection processes will be effective and sustainable in the long term.

The P-FBR was operated for 100 days in total at ambient 22 (±2) °C temperature, with a consistent 79% NO_3_^−^ removal efficiency to achieve the potable water limit of NO_3_^− ^< 50 mg L^−1^. Additionally, the denitrified effluent was disinfected electrochemically with 41.7 A h m^−3^ charge density and 0.4 kW h m^−3^ energy consumption. The combined treatment was realized without additional input of vitamins, or chemicals for pH control. The electrochemical cell operates solely on electricity, which can be generated from renewable sources such as solar or wind energy, which may allow location and electrical grid independency. Furthermore, the chlorination process takes place on-site, eliminating the need to purchase chemicals, as the Cl^−^ required to initiate disinfection is naturally present in the groundwater. The electrochemical unit could further enhance denitrification by making use of the H_2_ that is produced at the cathode. The H_2_ can serve as an additional electron donor for autotrophic denitrification and improve the overall NO_3_^−^ removal efficiency^48,49^.

Pathogens in drinking water have been linked to water-borne diseases, such as cholera, typhoid and diarrhea. An indication of the severity of the problem is the recent cholera outbrakes in Sub-Saharan African countries^50^. Exposure to high concentrations of nitrate in drinking water has been linked with infant respiratory problems and several other health issues^51^. Furthermore, the high chemical usage and dependence of current water treatment methods are of significant concern, particularly for landlocked countries. Approaches that can limit chemical use and provide chemical independence, where possible, are urgently required. In this regard, the combination of autotrophic FeS_2_-based denitrification with electrochemical disinfection could provide an inexpensive, renewable and small-footprint system for groundwater remediation, to comply with the SDG6.1.1 indicator for safe drinking water access that is free from fecal and chemical contamination, located on the premises and readily available.

## Methods

### Media and groundwater

Experiments in the P-FBR and EC reactors were conducted with synthetic (SGW) and real (GW) groundwater that was supplemented with nitrate (as NaNO_3_) (GW-Cycle I), chloride (as NaCl) (GW-Cycle II) and a higher concentration of total coliforms (TC) (GW-Cycle III) (Table 2). The SGW recipe was prepared according to the maximum values of previous studies with synthetic polluted groundwater and real polluted groundwater^28,52,53^ and contained: 0.005 g L^−1^ KNO_3_, 0.24 g L^−1^ NaNO_3_, 0.2 g L^−1^ MgSO_4_·7H_2_O, 0.043 g L^−1^ MgCl_2_·6H_2_O, 0.2 g L^−1^ CaCl_2_, 0.01 g L^−1^ NH_4_Cl, 0.1 g L^−1^ NaHCO_3_, 0.22 g L^−1^ NaCl and 0.02 g L^−1^ FeSO_4_·7H_2_O (178 mg NO_3_^−^ L^−1^ and 283 mg Cl^−^ L^−1^) (Supplementary Table 4).

**Table 2:** Operational scheme of the pyrite-based fluidized bed reactor (P-FBR) treating synthetic (SGW) and real (GW) groundwater.

Operational periods	HRT [h]	Operational time [d]
Batch 1	−	0–5
3× Batch	−	5–23
SGW-Cycle I	24	23–33
SGW-Cycle II	12	33–46
SGW-Cycle III	18	46–64
GW-Cycle I	18	64–80
GW-Cycle II	18	80–89
GW-Cycle III	18	89–100

*SGW* Synthetic groundwater.

GW-Cycle I: real groundwater with NaNO_3_ addition to final 178 mg NO_3_^−^ L^−1^ concentration.

GW-Cycle II: real groundwater with NaNO_3_ and NaCl addition to final 178 mg NO_3_^−^ L^−1^ and 283 mg Cl^−^ L^−1^ concentration.

GW-Cycle III: real groundwater with NaNO_3_, NaCl and total coliforms addition to final 178 mg NO_3_^−^ L^−1^, 283 mg Cl^−^ L^−1^ and 500 MPN 100 mL^−1^ total coliforms concentration.

Groundwater was sampled from two different privately owned wells in Co. Clare and Co. Galway in Ireland (characteristics presented in Supplementary Table 5) and was fridge stored at 4 °C till use as influent in the P-FBR. Although the groundwater sampled was contaminated with pathogens (Supplementary Table 5), the NO_3_^−^ and Cl^−^ concentrations were well below the concentrations that would be expected in a polluted groundwater source. Therefore, for subsequent experiments, real groundwater was used as the background solution and NO_3_^−^, Cl^−^ and TC were added in the respective cycles (Table 2) to reach 178 mg NO_3_^−^ L^−1^, 283 mg Cl^−^ L^−1^ and 500 MPN 100 mL^−1^ total coliforms in GW-Cycles I, II and III (Table 2).

### Pyrite-based denitrification experiments

**Fig. 6:**
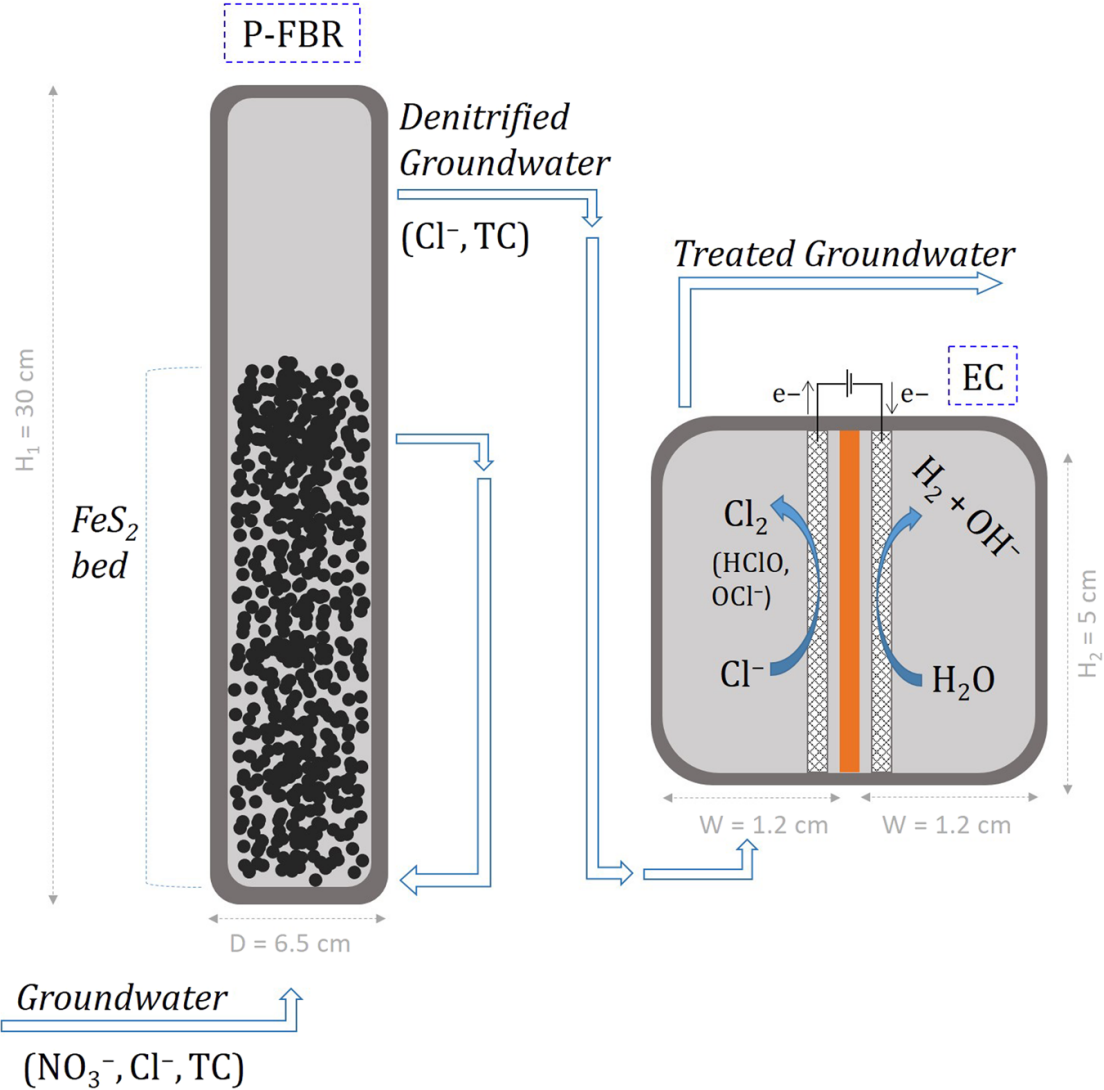
Schematic drawing of the combined pyrite-based denitrification reactor (P-FBR) and electrochlorination (EC) reactor for nitrate (NO_3_^−^) and total coliforms (TC) removal from groundwater (set-up picture in Supplementary Fig. 3). H_1_ and D are the height and the diameter of the P-FBR and H_2_ and W are the internal height and width of each of the two electrode chambers separated by the cation exchange membrane.

The fluidized bed denitrifying reactor (P-FBR) was a 6.5 cm diameter, 1 L glass column, as described in Carboni et al.^17^ (Fig. 6 and Supplementary Fig. 3a). The working liquid volume of the reactor, including recirculation piping, was 800 mL and the headspace was 200 mL. Two electrode probes were immersed in the liquid bed, for continuous monitoring of the ORP (MTC101, Hach^®^, Düsseldorf, Germany) and the pH (PHC101, Hach^®^, Düsseldorf, Germany) and were connected to portable HQ™ Series meters (Hach^®^, Düsseldorf, Germany). The headspace of the reactor was connected to a 1 L gas bag filled with 99.99% N_2_ gas to maintain anaerobic conditions in the reactor, as well as to a liquid displacement column to monitor excess production of N_2_ as the product of the denitrification process. A bed of approximately 10 cm was created at the bottom of the reactor by addition of 120 g FeS_2_ (99+% grade, 0.15–0.48 cm diameter, from Fischer Scientific, Hampton, USA) as described in Carboni et al.^17^. Fluidization was achieved with continuous upflow liquid recirculation by a peristaltic pump (323 S/D Watson Marlow, UK) at 200 mL min^−1^ allowing for a 30% – 40% bed expansion. The temperature in the reactor was not controlled, but it was monitored externally with a thermometer and it was stable at 22 (±2) °C.

Synthetic groundwater was used as medium and subsequent to the FeS_2_ addition, the reactor was flushed with 99.99% N_2_ through a gas bag to obtain anoxic conditions. Anoxic conditions were monitored with the ORP probe and were confirmed when the ORP dropped to values below 0 mV, as well as when the O_2_% in the headspace dropped to 2%. The reactor was then inoculated with 20% of the working volume (approximately 160 mL) with inoculum consisting of 60 mL of FeS_2_ biomass, directly obtained from a FeS_2_ denitrifying FBR, as described in Carboni et al.^17^ and 100 mL of the same biomass activated with S_2_O_3_^2−^ as electron donor in prior bottle incubations (data not shown). Initially, the reactor was operated in batch and after obtaining denitrification the reactor was switched to continuous operation, whereby the medium was supplied continuously from a 10 L bottle stored at 4 °C, with a peristaltic pump (Masterflex Cole-Parmer, Chicago, USA) according to the HRT required in every cycle (Table 2).

The denitrification performance of the P-FBR was evaluated based on the nitrate removal rate (in mg L^−1^ d^−1^, calculated as:

1 $${\rm{Nitrate\; removal\; rate}}=\frac{{\left[{{NO}}_{3}^{-}\right]}_{{eff}}-{\left[{{NO}}_{3}^{-}\right]}_{\inf }\,}{\frac{{HRT}}{24}}$$

where [NO_3_^−^]_eff_ and [NO_3_^−^]_inf_ are the effluent and influent concentrations of nitrate (in mg L^−1^) and HRT (in h) is the hydraulic retention time, that was 24, 12 and 18 h for the synthetic GW (SGW) experimental periods and 18 h for the experimental periods with the real groundwater (GW) (Table 2).

### Electrochemical disinfection experiments

All electrochemical experiments were conducted with a two compartment electrochemical cell (internal dimensions: 5 × 5 × 1.2 cm) allowing for an internal volume of 30 mL for each electrode compartment (Supplementary Fig. 3b). The inner and outer frames of the electrochemical cell were constructed from 4 PMMA transparent acrylic sheets with 1.2 cm thickness (Goodfellow, Hamburg, Germany). The two compartments were separated by a cation exchange membrane (CEM) (Fumasep© FKL-PK-130, Fumatech GmbH, Germany). Two platinum coated titanium (Pt/Ti) planar meshes (Redoxme AB, Norrköping, Sweden) with 5 × 5 cm projected surface were used as anode and cathode and two Ti rods (D = 3 mm) (Goodfellow, Hamburg, Germany) were used as current collectors. The Ti rods were laser welded under argon (Ar) gas shielding, perpendicular to the electrode surface (conducted by Dawnlough Ltd., Galway, Ireland) (Supplementary Fig. 3c). The two electrodes were positioned parallel to each other (distance between electrodes was ∼5 mm). A 3 M KCl Ag/AgCl electrode (BASi MF-2056, BASi, IN, USA, +0.210 V vs. SHE at 25 °C) was used as reference electrode (RE) in the anodic compartment and all reported potentials refer to this electrode. The electrochemical cell was controlled galvanostatically with a DC power supply (RS PRO Bench Power Supply, 150 W, 1 Output, 0 → 30 V, 0 → 5 A, RS Radionics, Dublin, Ireland) and electrochemical techniques were performed with a VSP potentiostat (Bio-Logic Science Instruments SAS, Seysinnet-Pariset, France). All current densities are reported with respect to the projected surface area of the anode (25 cm^2^).

Prior to cell operation, the compensated resistance between the anode and the reference electrode (R_an_, 80% compensation by potentiostat) and the cell resistance (R_cell_) were measured with the current interrupt (CI) method^54^ in 10 successive cycles (cycles of 50 ms at 100 mA followed by 50 ms open circuit with a recording period of 0.2 ms). The electrolyte used was SGW and GW according to the cycle that was tested every time and the resistances measured were 0.07 (±0.03) Ω for the anode and 17.83 (±3.83) Ω across the cell. The anode potential (E_WE_) and cell voltage (E_cell_) were monitored with the potentiostat by chronopotentiometry (CP).

The effluent of the denitrification reactor was treated in batch (1.2 L, total effluent of one day) in the anodic compartment of the electrochemical reactor, with an electrolysis time of 30, 60, 120, 180 min, depending on the current applied. In every batch test, the respective effluent of the P-FBR was used as anolyte (Table 3), while a 8.3 mM NaOH solution was used as catholyte (EC ≈ 2 mS cm^−1^), similar to the anolyte). Anolyte and catholyte were recirculated through the electrode compartments from 2 L bottles with a peristaltic pump (323 S/D Watson Marlow, Falmouth, UK) at 75 rpm. Samples for all chemical analyses were taken from the anolyte and catholyte recirculation lines.

**Table 3:** Operational scheme of the electrochemical reactor treating synthetic (SGW) and real (GW) P-FBR effluent.

Anolyte solution	Current applied [mA]	Replicates
SGW	50	3
	100	2
	150	1
GW-Cycle I	50	1
GW-Cycle II	50	1
	100	1
	150	1
GW-Cycle III	50	1
	100	1
	150	1

The charge density (in A h m^−3^) was calculated as:

2 $${\rm{Charge\; density}}=\frac{I\times {t}_{{electrolysis}}\,}{{V}_{{anolyte}}\,\times 60}$$

where I is the constant current applied (in mA), which was 50, 100 and 150 mA for the respective 2, 4 and 6 mA cm^−2^ current densities tested, t_electrolysis_ is the time of electrolysis batch (in min) and V_anolyte_ is the volume of the anolyte in every batch, which was 1.2 L.

The specific energy consumption (SECw in (kW h m^−3^) for the treatment of denitrified groundwater was calculated as:

3 $${\rm{SEC}}{\rm{w}}={ch}{arge}\,{density}\times {E}_{{cell}}$$

where E_cell_ is the cell voltage (in V), that was monitored during electrolysis.

### Analytical methods

Liquid samples taken from the influent and effluent of the P-FBR, as well as from the anolyte and catholyte of the electrochemical cell were filtered through a 0.22 μm membrane filter and were analyzed for NO_2_^−^, NO_3_^−^, SO_4_^2−^, S_2_O_3_^2−^, Cl^−^ and PO_4_^3−^ with a Dionex Aquion Ion Chromatography System (ThermoFisher Scientific, Waltham, USA), equipped with an IonPac AS14 A 4 × 250 mm column coupled to a AG14 A 4 × 50 mm guard column, running with a 3.03 mM NaHCO_3_/ 0.97 mM Na_2_CO_3_ eluent at 1 mL min^−1^ flow rate^55^. Total iron (Fe), K^+^, Na^+^, Mg^2+^ and Ca^2+^ were analyzed with an ICP-OES (ThermoFisher, Scientific Walthan, USA) operated at RF power: 1.2 kW, Ar plasma flow rate: 12 L min^−1^, auxiliary Ar flow rate: 1 L min^−1^ and nebulizer argon flow rate: 0.7 L min^−1^^17^. Ferrous iron (Fe^2+^), NH_4_^+^ (ppm) and alkalinity (mg L^−1^ as CaCO_3_) were measured with the colorimetric methods SM: 3500 Fe-B: Iron by Phenanthroline^56^, EPA-NERL: 350.1: (Rev. 2.0 1993): Ammonia by automated colorimetry^57^ and EPA 310.2 (Rev. 1974): Alkalinity by autoanalyser^58^, respectively, and analyzed photometrically with a Thermo Scientific™ Gallery™ automated analyzer (Thermo Fisher Scientific Oy, Vantaa, Finland), according to the manufacturer’s protocols. Ferric iron (Fe^3+^) concentrations were calculated as the difference between the measured Fe and Fe^2+^ concentrations. Electrical conductivity (EC) and pH were analyzed electrochemically with a Thermo Scientific™ Gallery™ analyzer equipped with a Gallery ECM unit with functional pH (984997) and reference electrodes (984996) (Thermo Fisher Scientific Oy, Vantaa, Finland), according to the manufacturer’s protocols.

Total carbon (TC) and inorganic carbon (IC) concentrations were measured with a TOC analyzer (TOC-L, Shimadzu, Kyoto, Japan) and the difference of the two was calculated as total organic carbon (TOC)^17^. Total nitrogen (TN) was determined photometrically with a SEAL Autoanalyzer AA3 3HR (SEAL Analytical Ltd, Wrexham, UK), according to Method No. G-157-96 Rev. 17 (Multitest MT17) (Hydrazine method) provided by the manufacturer. Gas samples taken from the headspace of the P-FBR were analyzed with a gas chromatograph (7890B, Agilent, Santa Clara, USA), equipped with a mass spectrometer (GC-MS) heated at 250 °C. Helium was used as the carrier gas with a flow rate of 2.5 mL min^−1^^17^.

### Chlorine analysis

Samples for free and total chlorine analysis were taken from the anolyte and catholyte recirculation lines and were measured immediately with a UV-1900 spectrophotometer with a temperature control chamber (Shimadzu, Kyoto, Japan) according to the standard DPD Colorimetric Method (4500 – Cl G)^59^. The resulting intensity was related to chlorine concentrations by means of a calibration curve (0.05–4 mg Cl_2_ L^−1^) constructed initially at 515 nm with KMnO_4_ standard solutions.

### Detection of total coliforms (TC) and *E. coli*

The Most Probable Number (MPN) of TC and *E. coli* was determined by the quanti-tray/2000 Colilert-18^®^ test (9223 B-2004 Colilert-18^®^)^60^ in 100 mL (MPN 100 mL^−1^) samples taken from the P-FBR influent and effluent as well as from the anolyte of the electrochemical cell. An IDEXX Quanti-tray sealer (IDEXX Laboratories Inc., Maine, USA) was used to seal the trays that were subsequently incubated for 22 h at 35 °C. For all dilutions required, filtered sterilized distilled water was used.

### Supplementary information

Ntagia_and_Lens_For_NPJCLEANWATER-00866R1_Supplementary_Information

## Data Availability

All data generated or analyzed during this study are included in this published article and its supplementary information files. Additional datasets generated during and/or analyzed during the current study are available from the corresponding author on request.
