# Supplementary material for: Pyrite-based denitrification combined with electrochemical disinfection to remove nitrate and microbial contamination from groundwater
Source: NPJ Clean Water. 2023 Aug 24;6(1):59. doi: 10.1038/s41545-023-00269-3 (PMC11041687; doi:10.1038/s41545-023-00269-3)
Supplement: Supplementary file 1 — Ntagia_and_Lens_For_NPJCLEANWATER-00866R1_Supplementary_Information [file 41545_2023_269_MOESM1_ESM.pdf]

Supplementary Information for:

Pyrite-based denitrification combined with electrochemical disinfection to remove nitrate and microbial contamination from groundwater

Eleftheria Ntagia* and Piet Lens

National University of Ireland, Galway, University Road, H91 TK33, Galway, Ireland

*Correspondence to: Department of Microbiology, School of Natural Sciences and Ryan Institute, NUI Galway, H91 TK33 Galway, Ireland;

E-mail address: eleftheria.ntagia@universityofgalway.ie; ele.ntagia@gmail.com

Keywords: groundwater, nitrate pollution, coliforms, electrochemical disinfection, electrochlorination

Supplementary Table 1 – Electrical conductivity (mS cm^{-1}), pH and SO_4^{2-} concentration (mg L^{-1}) in the influent and effluent of the P-FBR during operation with real groundwater (GW).

Operational cycle	HRT [h]	Influent			Effluent		
		EC [mS cm^{-1}]	pH	SO_4^{2-} [mg L^{-1}]	EC [mS cm^{-1}]	pH	SO_4^{2-} [mg L^{-1}]
GW-Cycle I	18	1.0 ± 0.1	~7	25.9 ± 3.4	0.9 ± 0.0	7.2 ± 0.0	27.8 ± 4.8
GW-Cycle II	18	1.9 ± 0.1	~7	25.7 ± 0.6	1.8 ± 0.2	7.3 ± 0.0	26.9 ± 1.5
GW-Cycle III	18	2.6 ± 0.5	~7	30.4 ± 3.1	2.7 ± 0.9	7.3 ± 0.0	28.1 ± 2.6

Supplementary Table 2 – Concentrations of cations (in mg L^{-1}) in the influent and effluent of the P-FBR during operation with real groundwater (GW). Iron species were not detected during continuous; P-FBR, GW operation.

Operational cycle	HRT [h]	Influent concentrations [mg L^{-1}]				Effluent concentrations [mg L^{-1}]			
		Ca^{2+}	Mg^{2+}	Na^{+}	K+	Ca^{2+}	Mg^{2+}	Na^{+-}	K+
GW-Cycle I	18	78.1 ± 6.0	17.3 ± 1.3	71.9 ± 8.0	1.0 ± 0.9	73.8 ± 6.3	18.1 ± 0.7	76.6 ± 7.1	2.1 ± 0.9
GW-Cycle II	18	68.2 ± 13.8	17.2 ± 1.3	256.1 ± 22.5	0.2 ± 0.3	71.2 ± 7.9	17.3 ± 0.3	233.9 ± 38.8	1.2 ± 0.6
GW-Cycle III	18	117.7 ± 20.9	17.0 ± 2.3	293.1 ± 17.0	3.6 ± 0.5	109.6 ± 19.4	18.2 ± 1.0	291.5 ± 13.2	5.4 ± 0.1

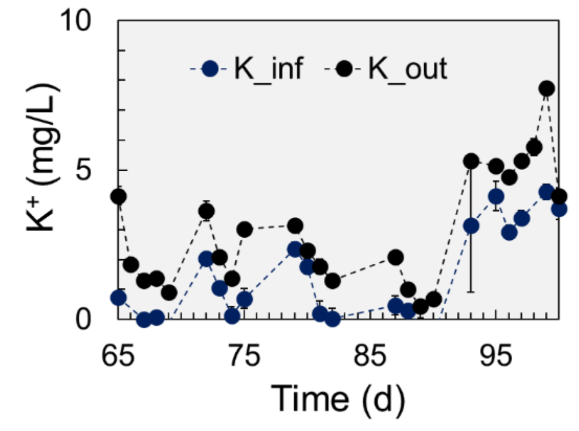

Supplementary Figure 1 – Potassium (K^+) concentration increase in the effluent of the P-FBR reactor, during operation with real groundwater (GW), between days 65 and 100.

Supplementary Table 3 – Effect of charge density on the production of free and total chlorine with synthetic (SGW) and real (GW) groundwater

	Chlorine (mg L^{-1}) produced with denitrified groundwater effluent			
	SGW		GW Cycle-II+III	
charge density (A h m^{-3})	free	total	free	total
41.67	0.169 ± 0.037	0.300 ± 0.006	0.043 ± 0.009	0.104 ± 0.006
83.33	0.245 ± 0.065	0.471 ± 0.121	0.115 ± 0.099	0.287 ± 0.121

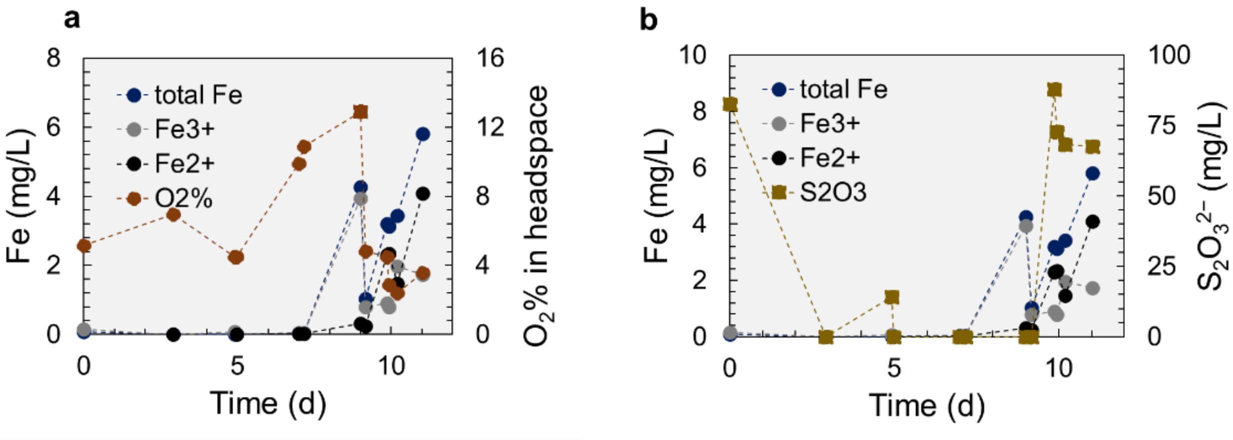

Supplementary Figure 2 – O_2 intrusion in the reactor and increase in measured Fe^{2+} and $S_2O_3^{2-}$ concentrations

Supplementary Methods

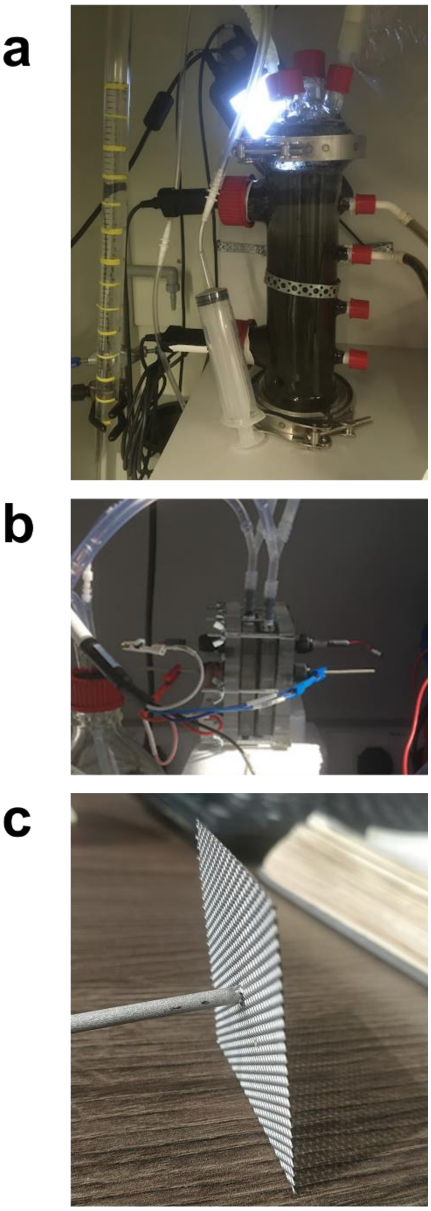

Supplementary Figure 3 – Combined continuous autotrophic denitrification with FeS2 as electron donor and electrochemical disinfection through Cl^- oxidation to Cl_2 ($HOCl$ + OCl^-) to treat groundwater. a: Pyrite-based denitrification reactor (P-FBR), b: Electrochemical cell for disinfection (electrochlorination reactor) c: Lazer welded Ti rod perpendicular to a Pt/Ti mesh. The resistance of the welded piece was measured with a multimeter from the tip of the rod to the top surface of each corner with the resistance ranging between 0.2 and 0.5 Ω and from the tip of the rod to its base which was 0.2Ω.

Supplementary Table 4- Synthetic groundwater, preparation for 1 L (adjusted from [1–3])

Chemical Compound	Concentration	Ion of interest	Concentration
KNO_3	0.005 g	NO_3^-	2.873 mM/ 178 mg L^{-1}
$NaNO_3$	0.24 g	Na^+	7.904 mM/ 182 mg L^{-1}
$MgSO_4 \cdot 7H_2O$	0.2 g	SO_4^{2-}	0.883 mM/
$MgCl_2 \cdot 6H_2O$	0.043 g	Mg^{2+}	1.023 mM/
$CaCl_2$	0.2 g	Ca^{2+}	1.802 mM/
NH_4Cl	0.01 g	NH_4^+	0.187 mM/
$NaHCO_3$	0.1 g	HCO_3^-	1.190 mM/
NaCl	0.22 g	Cl^-	7.995 mM/ 283 mg L^{-1}
$FeSO_4 \cdot 7H_2O$	0.02 g	Fe^{2+}	0.079 mM/

Supplementary Table 5 – Real groundwater characteristics from two wells. Total coliforms and *E. coli* are reported in the Results section in the main manuscript according to the real time measurements, as after storage in the fridge the total coliform and *E. coli* concentrations have dropped (pH value reported, measured with pH strips on-site).

Parameter/compound	Well 1	Well 2
pH	~ 7	~ 7
T (ºC)	14.00	12.00
Cl^- (mg L^{-1})	45.63 ± 0.24	30.96 ± 0.79
NO_2^- (mg L^{-1})	N.D.	N.D.
NO_3^- (mg L^{-1})	15.44 ± 0.66	5.42 ± 0.08
SO_4^{2-} (mg L^{-1})	27.79 ± 0.43	15.84 ± 0.37
NH_4^+ (mg L^{-1})	76.15 ± 62.21	14.12 ± 8.88
Alkalinity (mg L^{-1} as $CaCO_3$)	341.48 ± 42.79	223.89 ± 44.47
Conductivity (mS cm^{-1})	0.80 ± 0.02	0.49 ± 0.11
As (mg L^{-1})	N.D.	N.D.
Ca (mg L^{-1})	97.74 ± 3.63	59.50 ± 11.20
Cr (mg L^{-1})	N.D.	N.D.
Fe (mg L^{-1})	0.06 ± 0.00	0.06 ± 0.00

K (mg L^{-1})	3.73 ± 0.12	0.00 ± 0.00
Mg (mg L^{-1})	15.87 ± 0.09	12.87 ± 3.52
Mn (mg L^{-1})	0.02 ± 0.00	0.00 ± 0.00
Na (mg L^{-1})	19.55 ± 0.58	7.55 ± 2.25
Se (mg L^{-1})	N.D.	N.D.
TIC (mg L^{-1})	13.63 ± 2.16	9.05 ± 0.43
TOC (mg L^{-1})	75.25 ± 2.17	45.20 ± 0.86
Total coliforms (MPN 100 mL^{-1})	766.95 ± 153.85	31.80 ± 1.06
E. coli (MPN 100 mL^{-1})	250.05 ± 48.65	29.53 ± 1.72

Supplementary References

1. Wang, X., Prévoteau, A. & Rabaey, K. Impact of periodic polarization on groundwater denitrification in bioelectrochemical systems. *Environ. Sci. Technol.* **55**, 15371–15379 (2021).
2. Tammeaid, I. & Money, N. J. Elements of groundwater pollution and protection in a karst environment of Lusaka. in *International Mine Water Association Symposium 1993* 410–414 (International Mine Water Association (IMWA), 1993).
3. De Battisti, A., Formaglio, P., Ferro, S., Al Aukidy, M. & Verlicchi, P. Electrochemical disinfection of groundwater for civil use – An example of an effective endogenous advanced oxidation process. *Chemosphere* **207**, 101–109 (2018).
